# CST6 protein and peptides inhibit breast cancer bone metastasis by suppressing CTSB activity and osteoclastogenesis

**DOI:** 10.7150/thno.62187

**Published:** 2021-10-11

**Authors:** Xiaoxun Li, Yajun Liang, Cheng Lian, Fangli Peng, Yansen Xiao, Yunfei He, Chengxin Ma, Yuan Wang, Peiyuan Zhang, Yuhan Deng, Yunpeng Su, Cheng Luo, Xiangyin Kong, Qingcheng Yang, Tong Liu, Guohong Hu

**Affiliations:** 1 Department of Breast Surgery, Harbin Medical University Cancer Hospital, Harbin, China.; 2 CAS Key Laboratory of Tissue Microenvironment and Tumor, Shanghai Institute of Nutrition and Health, University of Chinese Academy of Sciences, Chinese Academy of Sciences, Shanghai, China.; 3 Department of Orthopedics, Shanghai Jiao Tong University Affiliated Sixth People's Hospital, Shanghai, China.; 4 Novomab Biopharmaceuticals Inc., Nanjing, China.; 5 Drug Discovery and Design Center, State Key Laboratory of Drug Research, Shanghai Institute of Materia Medica, Chinese Academy of Sciences, Shanghai, China.

**Keywords:** Breast cancer, Bone metastasis, Osteoclastogenesis, CST6, Peptide drug

## Abstract

**Background:** Bone metastasis is a frequent symptom of breast cancer and current targeted therapy has limited efficacy. Osteoclasts play critical roles to drive osteolysis and metastatic outgrowth of tumor cells in bone. Previously we identified CST6 as a secretory protein significantly downregulated in bone-metastatic breast cancer cells. Functional analysis showed that CST6 suppresses breast-to-bone metastasis in animal models. However, the functional mechanism and therapeutic potential of CST6 in bone metastasis is unknown.

**Methods:** Using *in vitro* osteoclastogenesis and *in vivo* metastasis assays, we studied the effect and mechanism of extracellular CST6 protein in suppressing osteoclastic niches and bone metastasis of breast cancer. A number of peptides containing the functional domain of CST6 were screened to inhibit bone metastasis. The efficacy, stability and toxicity of CST6 recombinant protein and peptides were evaluated in preclinical metastasis models.

**Results:** We show here that CST6 inhibits osteolytic bone metastasis by inhibiting osteoclastogenesis. Cancer cell-derived CST6 enters osteoclasts by endocytosis and suppresses the cysteine protease CTSB, leading to up-regulation of the CTSB hydrolytic substrate SPHK1. SPHK1 suppresses osteoclast maturation by inhibiting the RANKL-induced p38 activation. Importantly, recombinant CST6 protein effectively suppresses bone metastasis *in vitro* and *in vivo*. We further identified several peptides mimicking the function of CST6 to suppress cancer cell-induced osteoclastogenesis and bone metastasis. Pre-clinical analyses of CTS6 recombinant protein and peptides demonstrated their potentials in treatment of breast cancer bone metastasis.

**Conclusion:** These findings reveal the CST6-CTSB-SPHK1 signaling axis in osteoclast differentiation and provide a promising approach to treat bone diseases with CST6-based peptides.

## Introduction

Distant metastasis is the major cause of mortality of patients with breast cancer 1. Skeleton is the most common site where breast cancer cells tend to metastasize 2. Normally, the rigid bone matrix limits the growth of tumor cells; however, bone is also a rich reservoir of nutrients and growth factors to support tumor growth through the vicious circle of osteolytic metastasis. Disseminated tumor cells produce soluble factors, such as receptor activator of nuclear factor kappa-B ligand (RANKL), parathyroid hormone-related peptide (PTHrP), interleukin-6, Jagged1, and matrix metalloproteases, to stimulate osteoclast maturation. In turn enhanced osteoclast activity promotes tumor cell survival and proliferation by releasing nutrients and growth factors embedded in bone matrix 3-5. Thus, targeting osteoclasts represents a major approach to interrupt this osteolytic vicious cycle and stop bone metastasis. Indeed, many of the current therapeutic drugs of bone metastasis, including bisphosphonates and the anti-RANKL antibody Denosumab, are osteoclast-targeting agents. However, the clinical benefit of these agents is limited by side effects, high cost or minimal long-term benefit 6, 7. Development of additional therapeutic strategies based on new understanding of the communication between tumor cells and bone stroma is of paramount clinical significance.

CST6 belongs to the type 2 cystatin family, which are mainly extracellular polypeptide inhibitors of cysteine proteases to prevent extra proteolysis 8. Three proteases, including Cathepsin B (CTSB), Cathespin L (CTSL) and Legumain (LGMN), are known to be inhibited by CST6 in human cells. The CST6 protein consists of only 149 amino acids including a 28-residue signal peptide. Epigenetic silencing of *CST6* has been widely observed in multiple cancer types 9-14. Previously by secretomic profiling, we found that CST6 is downregulated in bone-tropic breast cancer cells and tumor samples. Functional analysis validated the suppressing roles of CST6 in tumor invasion and breast-to-bone metastasis 15. However, the molecular mechanism of CST6 in metastasis regulation, as well as the clinical potential of CST6 for prognosis and treatment of metastasis, remains unknown.

## Results

### CST6 suppresses osteoclastogenesis and bone colonization of breast cancer cells

**Figure 1:**
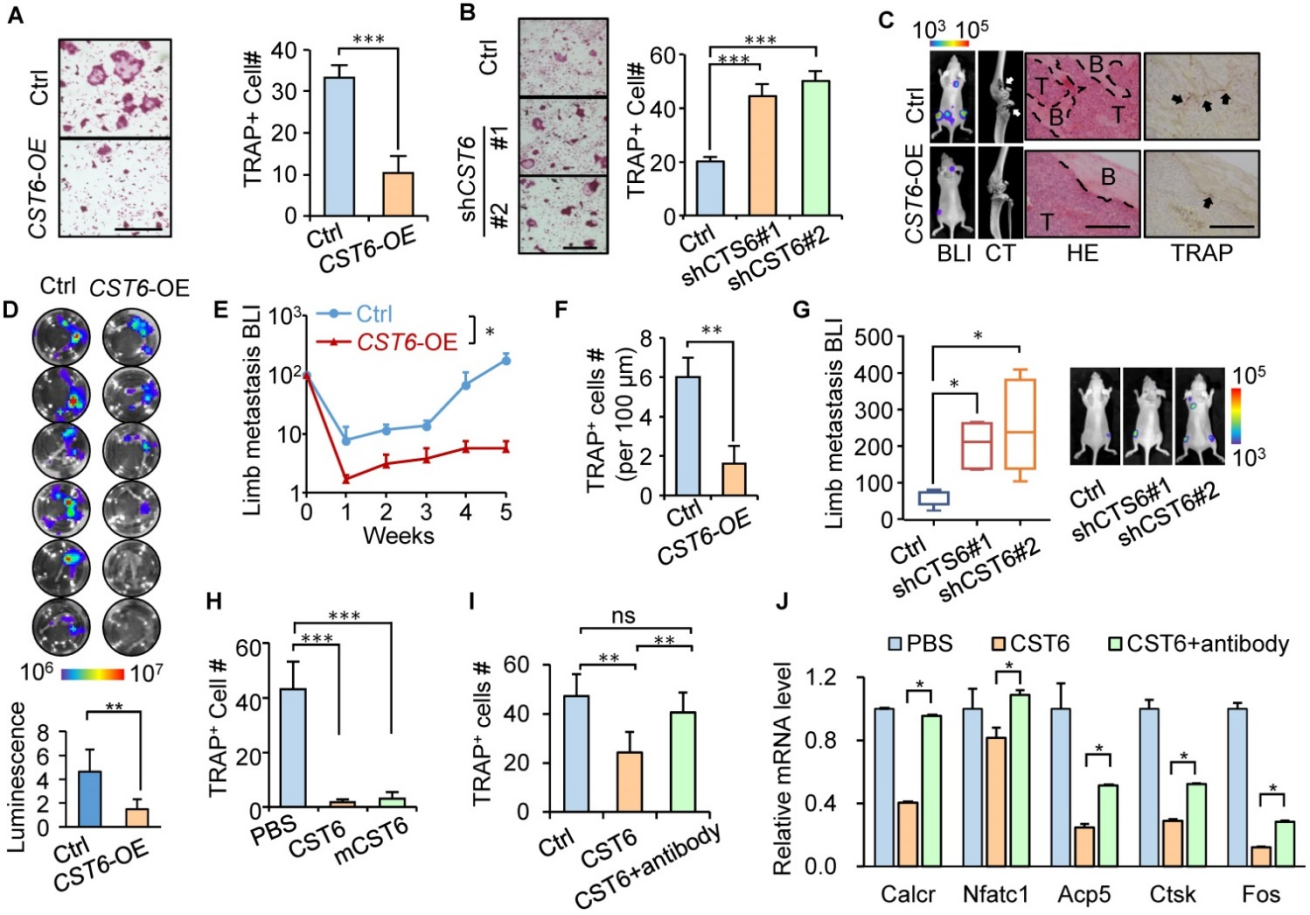
** CST6 suppresses osteoclastogenesis and bone metastasis.** (**A**) Osteoclastogenesis of murine primary bone marrow cells with conditioned medium (CM) from *CST6*-overexpressing or control SCP2 (n = 3 independent experiments). Ctrl, control. (**B**) Osteoclastogenesis of primary bone marrow culture with CM from CST6 knockdown or control SCP4 cells (n = 3 independent experiments). (**C-F**) Intracardiac injection of SCP2 cells with CST6 overexpression for bone metastasis analysis (n = 10 mice per group). Shown are representative bioluminescence imaging (BLI), micro-CT, H&E and TRAP staining of the hind legs (**C**), ex vivo BLI analysis of hind legs of the mice (**D**), BLI quantitation of limb metastasis (**E**) and quantitation of TRAP^+^ cells along the tumor-bone interface (**F**). White and black arrows (**C**) point to the area of bone damage and osteoclasts, respectively. Scale bar, 200 μm. (**G**) Intracardiac injection of SCP4 cells with *CST6* knockdown for bone metastasis analysis. (**H**) Osteoclastogenesis of primary bone marrow cells treated with 32 nM human or mouse (mCST6) recombinant CST6 proteins. (**I**) Osteoclastogenesis of RAW264.7 cells cultured with RANKL and treated with 32 nM human CST6 protein or 32 nM human CST6 and 7 µg/ml CST6 neutralizing antibody. (**J**) Expression of various gene markers of osteoclast differentiation in RAW264.7 cells treated with CST6 recombinant protein or CST6 recombinant protein and CST6 neutralizing antibody (n = 3 biological repeats). Scale bar, 150 μm. *P < 0.05, **P < 0.01, ***P < 0.001; ns, not significant.

Previously we showed that CST6 suppresses bone metastasis of breast cancer and observed a reduction of osteoclasts in the metastases caused by *CST6*-expressing cancer cells 15. To further assess the effect of tumor-derived CST6 on osteoclastogenesis, *CST6* was overexpressed in SCP2, a bone-tropic breast cancer cell line 16 with weak expression of *CST6* 15. *CST6* overexpression enhanced the extracellular level of the protein in conditioned medium (CM) of SCP2 (Fig. S1A). Osteoclastogenesis analysis by culturing primary bone marrow of mice with SCP2 CM showed that *CST6* overexpression greatly diminished CM-induced osteoclast maturation from bone marrow cells, as showed by tartrate-resistant acid phosphatase (TRAP) staining (Fig. 1A). In contrast, *CST6* knockdown with two different short-hairpin RNAs in the weakly bone-metastatic breast cells SCP4 16 reduced the secreted level of CST6 (Fig. S1B) and enhanced the capacity of SCP4 CM to induce osteoclastogenesis of bone marrow cells (Fig. 1B). Importantly, xenograft metastasis analyses by intracardiac injection of these cancer cells into mice showed that CST6 significantly reduced the number of TRAP^+^ osteoclasts along the tumor-bone interface and inhibited bone matrix destruction, leading to suppression of bone metastasis (Fig. 1C-G). We further found that CTS6 was expressed and secreted only by tumor cells, but not by osteoclasts derived from primary bone marrow or RAW264.7 cells (Fig. S1C). These data validated a role of tumor-derived CST6 to regulate osteoclasts in the bone microenvironment for bone metastasis.

To elucidate the direct cell target of extracellular CST6 for osteoclast regulation, we expressed and purified the human CST6 recombinant protein, followed by treating murine bone marrow cells cultured in RANKL-containing medium with the CST6 protein for osteoclastogenesis assay. The result showed that human recombinant CST6 significantly suppressed RANKL-induced differentiation of bone marrow cells into osteoclasts in a dosage-dependent manner (Fig. 1H and Fig. S1D), but did not affect the viability of osteoclasts (Fig. S1E). The murine recombinant CST6 protein showed a similar inhibitory effect on osteoclastogenesis (Fig. 1H). We further used the pre-osteoclast RAW264.7 cell line for osteoclastogenesis analysis and also observed the inhibition of osteoclastic differentiation of cells by recombinant CST6 protein (Fig. 1I). Several marker genes of osteoclastic differentiation, including *Caclr*, *Nfatc1*, *Acp5*, *Ctsk* and *Fos*, were also downregulated by CST6 (Fig. 1J). Furthermore, the suppression of osteoclastogenesis by recombinant CST6 could be rescued by a CST6 neutralizing antibody (Fig. 1I, J). Notably, CST6 protein did not directly affect the growth of tumor cells (Fig. S1F). Collectively, these data indicated that CST6 directly targets on cells of osteoclast lineage, instead of tumor cells, to regulate osteoclastogenesis.

### CST6 inhibits CTSB enzymatic activity for osteoclast suppression

**Figure 2:**
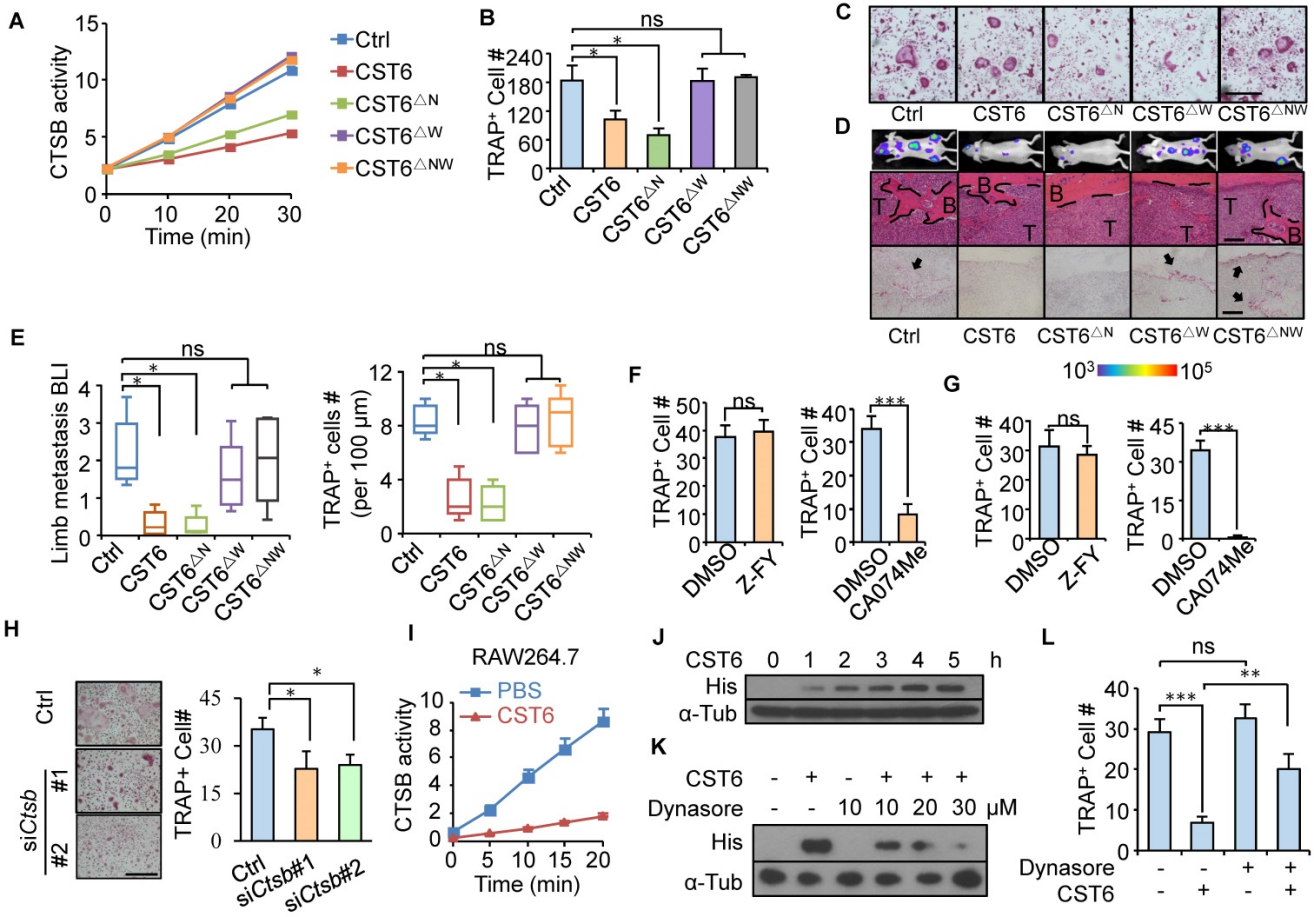
** CST6 regulates osteoclastogenesis by inhibiting CTSB.** (**A**) CTSB enzymatic activity in lysates of SCP2 cells expressing wild type or mutant CST6 (n = 3 independent experiments). ∆N, N64A; ∆W, W135A; ∆NW, N64A and W135A double mutant. (**B, C**) Osteoclastogenesis of primary bone marrow treated with CM from SCP2 overexpressing CST6 mutants (n = 3 independent experiments). Representative images of TRAP staining were shown in **C**. Scale bar, 150 μm. (**D, E**) *In vivo* bone metastasis analysis of SCP2 cells expressing CST6 mutants (n = 10 mice per group). Shown are representative images of BLI, H&E and TRAP staining of bone metastases in hind legs at week 6 after SCP2 injection (**D**), and quantitation of BLI signal and TRAP^+^ cells (**E**). Scale bar, 200 μm. (**F**) Osteoclastogenesis of primary bone marrow treated with 10 μM Z-FY(t-Bu)-DMK (Z-FY) or CA-074Me (n = 3 independent experiments). (**G**) RAW264.7 osteoclastogenesis after treatment with Z-FY or CA074Me (n = 3 independent experiments). (**H**) Osteoclastogenesis of murine primary bone marrow cells transfected with *Ctsb* siRNA. (**I**) Intracellular CTSB activity of RAW264.7 after treatment with 32 nM CST6 protein. (**J**) RAW264.7 was cultured with recombinant His-tagged CST6 protein for the indicated time, and intracellular CST6-His level was analyzed by Western blots after PBS washing of the cells. α-Tub, α-Tubulin. (**K**) Western blot analysis of intracellular CST6-His level of RAW264.7 after culturing the cells with CST6-His protein and various concentrations of Dynasore. (**L**) RAW264.7 osteoclastogenesis after CST6 protein and Dynasore treatment. Scale bar, 150 μm. *P < 0.05, **P < 0.01, ***P < 0.001; ns, not significant.

CST6 binds to and inhibits cathepsins (CTSB and CTSL) and LGMN through distinct binding sites of the protein 17. To interrogate the protease target of CST6 for osteoclastogenesis regulation, we cloned the N64A (CST6^△N^) and W135A (CST6^△W^) point mutations of CST6 protein (Fig. S2A) that were known to selectively diminish the inhibitory effect of CST6 on LGMN and cathepsins 17. Enzymatic activity assays confirmed that CST6^△N^ and CST6^△W^ could only inhibit cathepsins and LGMN, respectively, while the double mutant CST6^△NW^ failed to inhibit either protease (Fig. 2A and Fig. S2B). Notably, CST6^△W^ and CST6^△NW^ completely lost the capacity to suppress osteoclastogenesis, while CST6^△N^ remained as an inhibitor of osteoclast maturation (Fig. 2B, C). *In vivo* metastasis studies also showed that the W135A mutation, but not the N64A mutation, diminished the inhibitory effect of CST6 on osteoclasts and bone metastasis (Fig. 2D, E). These results indicated that CTSB or CTSL was the downstream target of CST6 to suppress osteoclastogenesis. Further, we found that the selective CTSB inhibitor CA-074Me, but not the CTSL inhibitor Z-FY(t-Bu)-DMK, was able to inhibit osteoclast differentiation of primary bone marrow cells and RAW264.7 cells (Fig. 2F, G, and Fig. S2C-E). In addition, *Ctsb* knockdown in murine primary bone marrow cells with siRNAs also significantly depressed osteoclastogenesis (Fig. 2H). Taken together, these suggested that CTSB suppression mediates the role of CST6 in osteoclastogenesis.

Cathepsins are lysosomal proteases and function only intracellularly in acidic environment 18. To verify that extracellularly derived CST6 could inhibit the intracellular CTSB activity of osteoclasts, RAW264.7 cells were cultured in the medium containing recombinant CST6 protein and the cell lysates were collected for CTSB activity analysis after phosphate-buffered saline (PBS) washing of the cells. The results showed that CST6 treatment indeed inhibited the intracellular CTSB activity of RAW264.7 (Fig. 2I). More importantly, gradual accumulation of the CST6 protein inside RAW264.7 cells was observed following extracellular CST6 treatment (Fig. 2J and Fig. S3A). Since osteoclasts are active in endocytosis, it is likely that exogenous CST6 is transported into osteoclasts via endocytosis. Indeed, we observed a co-localization of CST6 that entered the cells with the lysosome marker LAMP1 (Fig. S3B). More importantly, treating RAW264.7 with the endocytosis inhibitor Dynasore reduced the intracellular accumulation of CST6 in a dosage-dependent manner (Fig. 2K and Fig. S3C). Endocytosis inhibition also rescued the CST6-suppressed osteoclastogenesis of RAW264.7 cells (Fig. 2L). These findings suggested that extracellular CST6 protein could be internalized by pre-osteoclasts through endocytosis to inhibit CTSB and osteoclast differentiation.

### CST6 stabilizes SPHK1 and inhibits p38 in osteoclasts

**Figure 3:**
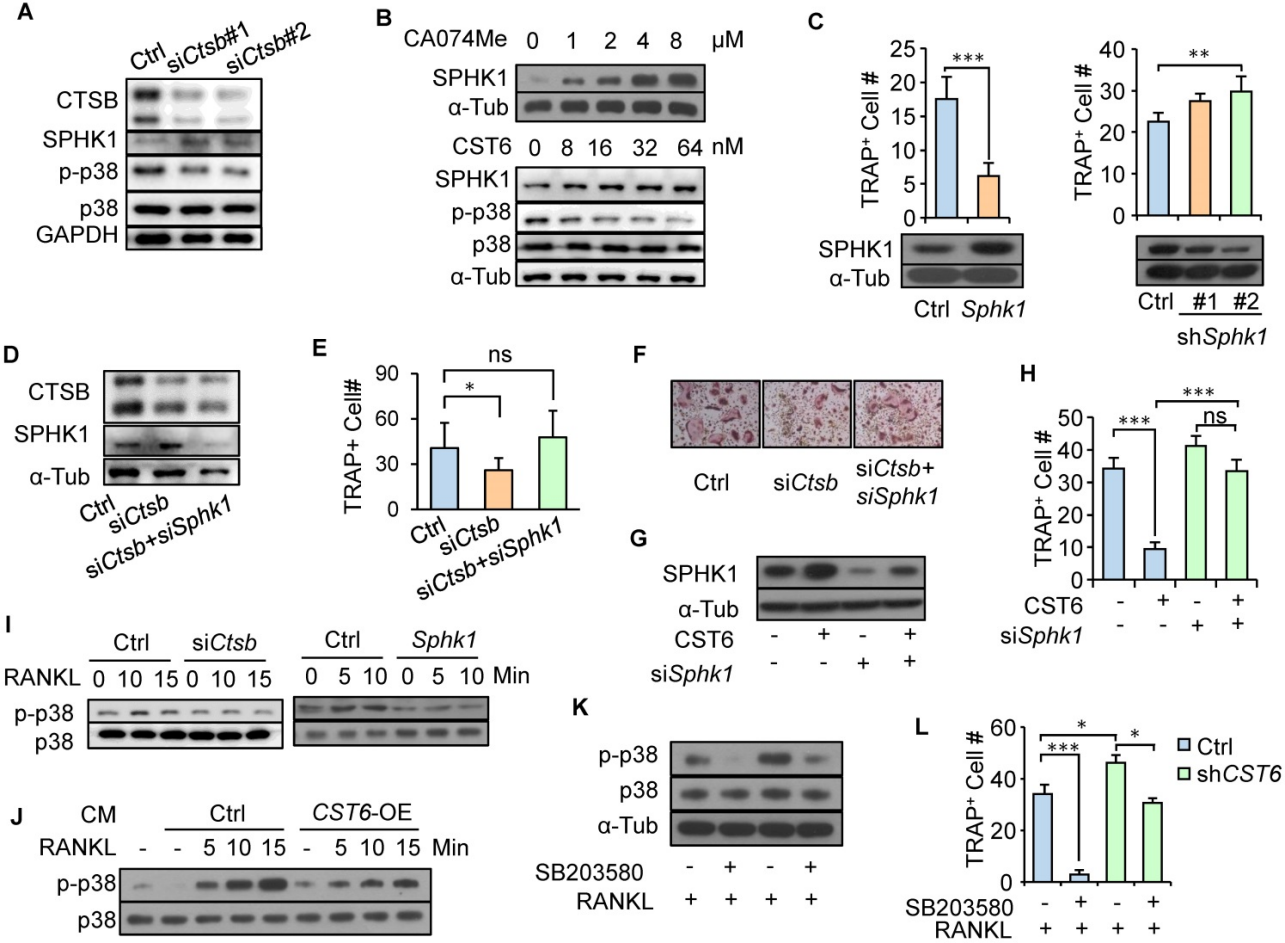
** CST6 regulates osteoclastogenesis by stabilizing SPHK1 and inhibiting p38.** (**A**) SPHK1 and phosphorylation of p38 in murine primary bone marrow cells transfected with *Ctsb* siRNA. (**B**) SPHK1 and phosphorylation of p38 in RAW264.7 after treatment with CA074Me or CST6 protein. (**C**) RAW264.7 osteoclastogenesis after *Sphk1* overexpression and* Sphk1* knockdown. (**d-f**) SPHK1 expression (**D**) and osteoclastogenesis (**E, F**) of murine primary bone marrow cells with *Ctsb* and *Sphk1* knockdown. (**G, H**) SPHK1 expression (**G**) and osteoclastogenesis (**H**) of RAW264.7 after *Sphk1* knockdown and recombinant CST6 treatment. (**I**) Phosphorylation of p38 in primary bone marrow cells with *Ctsb* knockdown (left) and in RAW264.7 cells after *Sphk1* knockdown (right), together with RANKL treatment of various time. (**J**) Phosphorylation of p38 in RAW264.7 cells treated with CM from *CST6*-ovexpressing or control SCP2 cells, together with RANKL treatment of various time. (**K, L**) Phosphorylation of p38 (**K**) and osteoclastogenesis (**L**) of RAW264.7 treated with RANKL, SB203580 and CM from *CST6*-knockdown or control SCP4 cells. *P < 0.05, **P < 0.01, ***P < 0.001; ns, not significant.

To further elucidate the downstream mechanism of CTSB in osteoclast regulation, we searched the peptidase database MEROPS 19 and found that sphingosine kinase 1 (SPHK1) was shown to be one of the CTSB substrates. SPHK1 is cleaved by CTSB 20, and more importantly, SPHK1 can negatively regulate osteoclast differentiation by phosphorylating sphingosine into sphingosine-1-phosphate (S1P) 21. Concordantly, we observed that *Ctsb* knockdown increased SPHK1 expression in primary bone marrow cells (Fig. 3A). Treating RAW264.7 cells with the CST6 protein or the CTSB inhibitor CA-074Me or stabilized the intracellular SPHK1 protein in a dose-dependent manner (Fig. 3B). In addition, *Sphk1* overexpression in RAW264.7 significantly inhibited differentiation of the cells into osteoclasts, while *Sphk1* knockdown displayed an opposite effect (Fig. 3C). Notably, simultaneous *Sphk1* and *Ctsb* knockdown in primary bone marrow cells (Fig. 3D) reversed the suppression of osteoclastogenesis caused by *Ctsb* inhibition (Fig. 3E, F). Similarly, *Sphk1* knockdown could also recover osteoclastogenesis of RAW264.7 that was suppressed by the treatment of CST6 recombinant (Fig. 3G, H). These results indicated that CST6 attenuates osteoclastogenesis by upregulating SPHK1.

Previous studies showed that RANKL-induced p38 signaling activation is critical for osteoclast maturation 22 and SPHK1 inactivates p38 21. We also observed that RANKL treatment led to p38 phosphorylation in pre-osteoclasts, while *Ctsb* knockdown and *Sphk1* overexpression in pre-osteoclasts blocked such effect of RANKL and inhibited p38 phosphorylation (Fig. 3I). Furthermore, treating pre-osteoclasts with CM from* CST6*-overexpressing cancer cells also diminished RANKL-induced p38 phosphorylation in pre-osteoclasts (Fig. 3J). CST6 treatment also inhibited p38 activation (Fig. 3B). To further validate the involvement of p38 signaling in CST6-suppresesed osteoclastogenesis, the p38 inhibitor SB203580 was used to treat RAW264.7 cells that were cultured in cancer cell CM. It was found that *CST6* knockdown in cancer cells enhanced CM-induced p38 phosphorylation and osteoclast maturation of RAW264.7, while SB203580 treatment showed the opposite effect (Fig. 3K, L). Taken together, our data showed that CST6 inhibits CTSB activity and stabilizes SPHK1, leading to suppression of p38 activation and osteoclast maturation.

### Recombinant CST6 protein suppresses breast cancer bone metastasis

**Figure 4:**
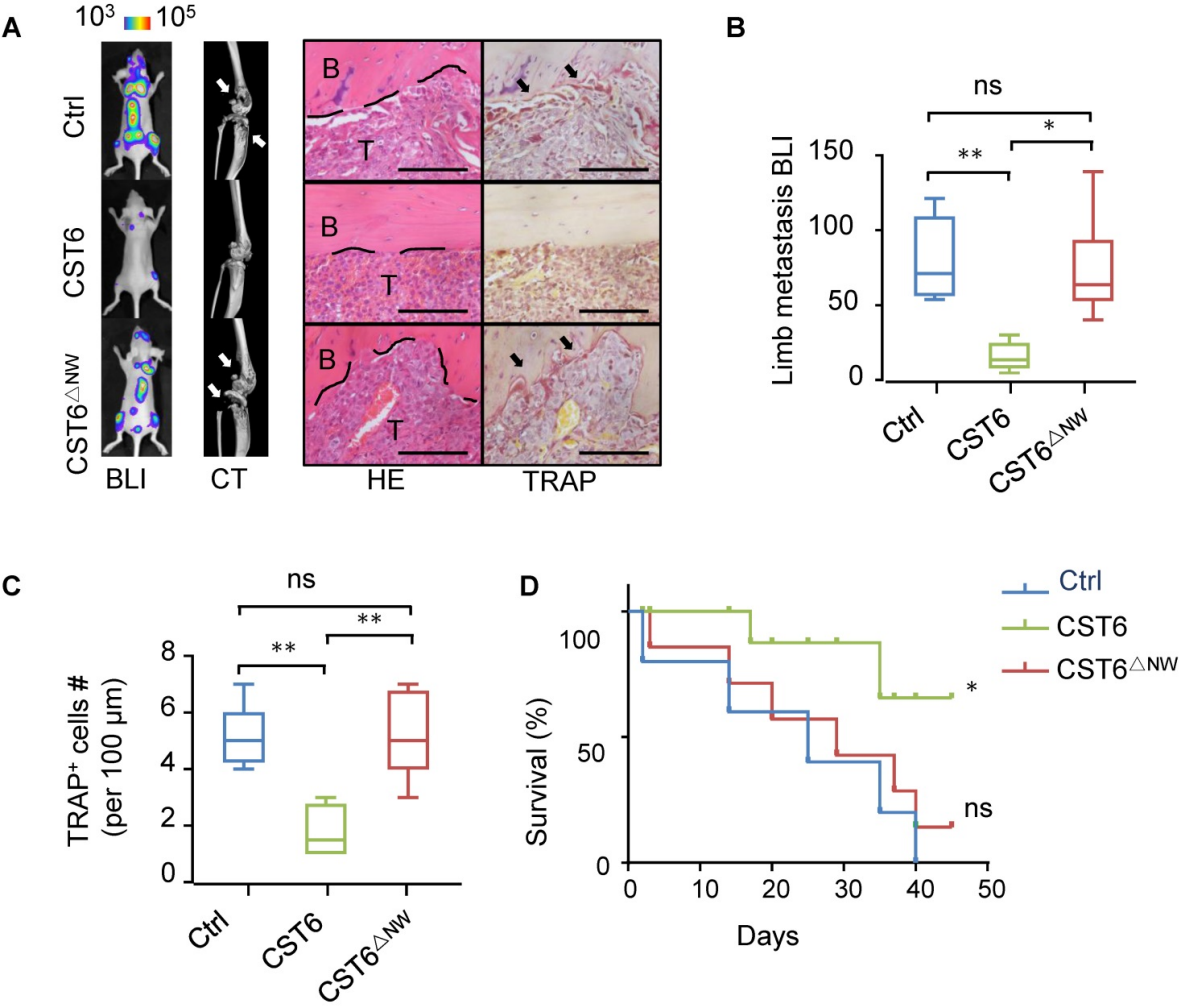
** CST6 recombinant protein effectively inhibits bone metastasis of breast cancer in mice.** (**A**) Representative BLI, micro-CT, H&E and TRAP staining images of SCP2 bone metastases at week 5 after treatment of 1 mg/kg/day wild type or mutant CST6 proteins (n = 10 mice per group). Scale bar, 200 μm. (**B, C**) BLI (**B**) and osteoclast (**C**) quantitation of the metastasis. (**D**) Survival analysis of the mice. *P < 0.05, **P < 0.01; ns, not significant.

Next, we tested whether the recombinant CST6 protein could be used to treat bone metastasis of breast cancer. Nude mice were pre-inoculated with SCP2 cancer cells and then treated with 1 mg/kg CST6 recombinant protein by intravenous delivery every day. The recombinant mutant protein CST6^△NW^ was used as a negative control. In accordance with the *in vitro* function of CST6 on osteoclasts, CST6 treatment efficiently alleviated bone metastasis (Fig. 4A, B), reduced osteoclastogenesis and bone destruction (Fig. 4A, C), and extended survival of the mice (Fig. 4D). These data argued for the potential of CST6 as a protein drug to treat bone metastatic disease.

### CST6 peptides suppress osteoclastogenesis and breast cancer bone metastasis

**Figure 5:**
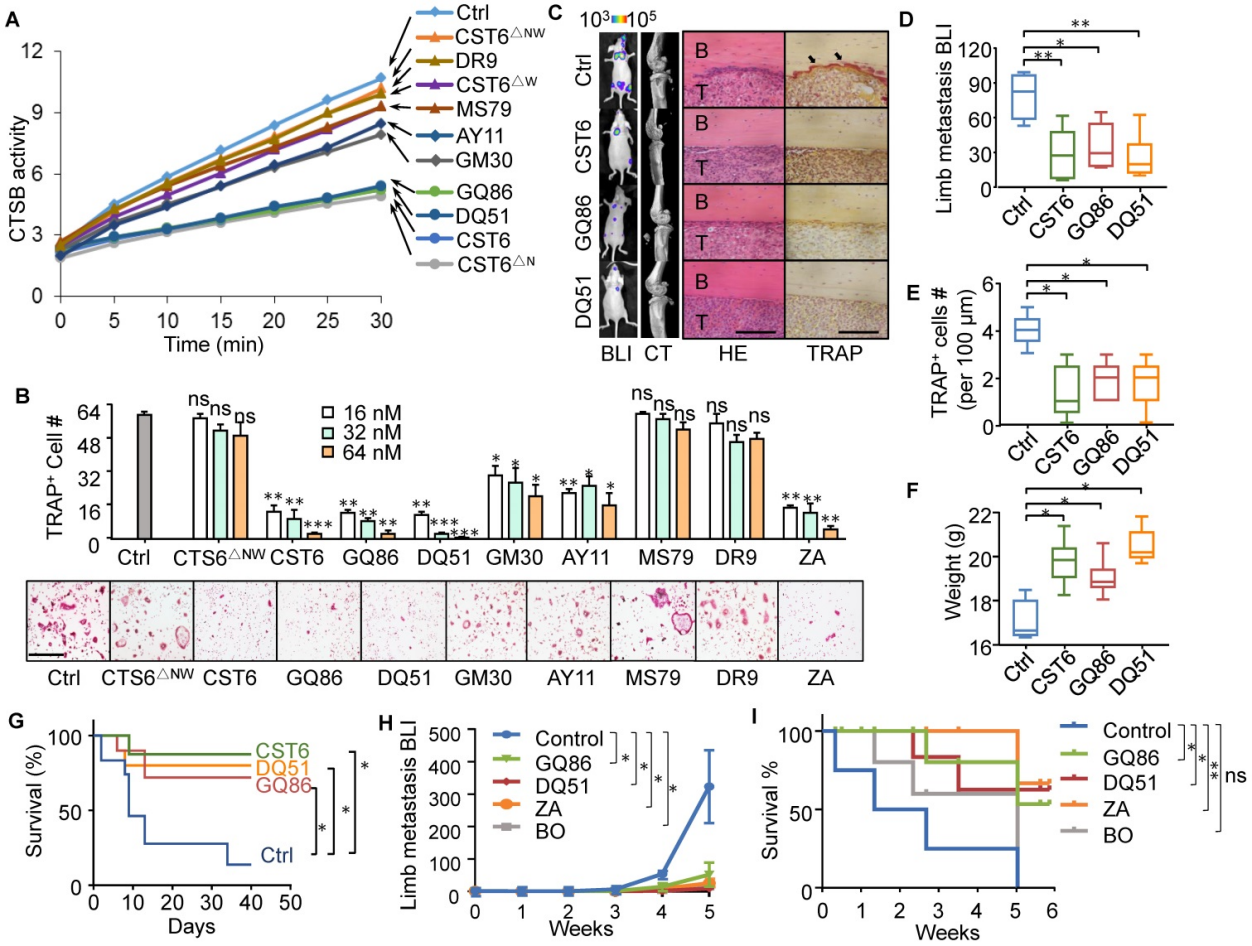
** CST6 peptides inhibit osteoclastogenesis and bone metastasis of breast cancer.** (**A**) The effects of CST6 peptides to inhibit CTSB activity of RAW264.7 lysate (n = 3 independent experiments). (**B**) Osteoclastogenesis of primary bone marrow (n = 3 independent experiments) treated CST6 peptides or Zoledronic Acid (ZA). Scale bar, 150 μm. Asterisks indicate significance compared to the control. (**C-G**) The effects of CST6 peptides to inhibit SCP2 bone metastasis in mice after treatment of 1 mg/kg/day CST6 peptides (n = 10 mice per group). Shown are representative BLI, micro-CT, H&E and TRAP staining images of bone metastases in hind legs at week 5 (**C**), BLI quantitation of metastasis (**D**), TRAP^+^ cell quantitation in metastases (**E**), body weights (**F**) and survival analysis (**G**) of the mice. Scale bar, 200 μm. (**H, I**) SCP2 bone metastasis in nude mice (**H**) and animal survival (**I**) with treatment of 1 mg/kg/day CST6 peptides, 1 mg/kg/day Zoledronic Acid (ZA) or 1 mg/kg/day Bortezomib (BO). *P < 0.05, **P < 0.01, ***P < 0.001; ns, not significant.

Peptides have promising potentials in clinical applications. Thus, we wanted to screen for CST6-mimicking peptides with smaller molecular sizes as candidate drugs of bone metastasis. The protein structure of CST6 has been well studied and it is reported that a hairpin loop containing the highly conserved Gln-Leu-Val-Ala-Gly (QLVAG) fragment (Fig. S4A, B) was important for CST6 to bind to cathepsins for enzymatic inhibition 23-27. Thus, we designed a series of peptides of various lengths that include the QLVAG fragment or other CST6 domains (Fig. S4A; Table S1). These candidate peptides were produced by recombinant expression (Fig. S4C) or chemical synthesis. We tested their capabilities to inhibit CTSB enzymatic activity and osteoclastogenesis in comparison to CST6 wild type and mutant proteins. It was found that two QLVAG-containing peptides GQ86 and DQ51, in lengths of 86 and 51 amino acids, respectively, significantly suppressed CTSB (Fig. 5A) and inhibited osteoclastogenesis of bone marrow cells with dosage-dependent effects similar to that of the full length CST6 protein (Fig. 5B). In addition, the osteoclast-suppressing performance of these recombinant protein and peptides was similar to or better than Zoledronic Acid (Fig. 5B), a bisphosphonate that is in clinical use to treat bone metastasis and osteoporosis. Other shorter QLVAG-containing peptides such as GM30 and AY11 also displayed moderate CTSB and osteoclast-suppressing activity. In contrast, peptides without the QLVAG fragment in various lengths failed to inhibit CTSB or osteoclastogenesis (Fig. 5A, B).

We further assessed the *in vivo* efficacy of the peptides to inhibit bone metastasis of SCP2 cells in the mice. Both GQ86 and DQ51 significantly inhibited bone metastasis and osteoclast maturation at the treatment concentration of 1 mg/kg, with the efficacy similar to that of full length CST6 (Fig. 5C-E). The peptide treatment also restored the body weight and extended survival of the animals (Fig. 5F, G). We further compared the peptides to two clinical drugs for osteolytic bone lesion, Zoledronic Acid and Bortezomib. The data showed that the efficacy of the peptides was similar to both drugs for inhibition of bone metastasis and extension of animal survival (Fig. 5H, I, and Fig. S5A). In addition, the peptides, but not Bortezomib, recovered the body weight of mice that was reduced due to metastasis (Fig. S5B), indicating less toxicity of the peptide than Bortezomib.

### Pharmacological evaluation of CST6 protein and peptides

**Figure 6:**
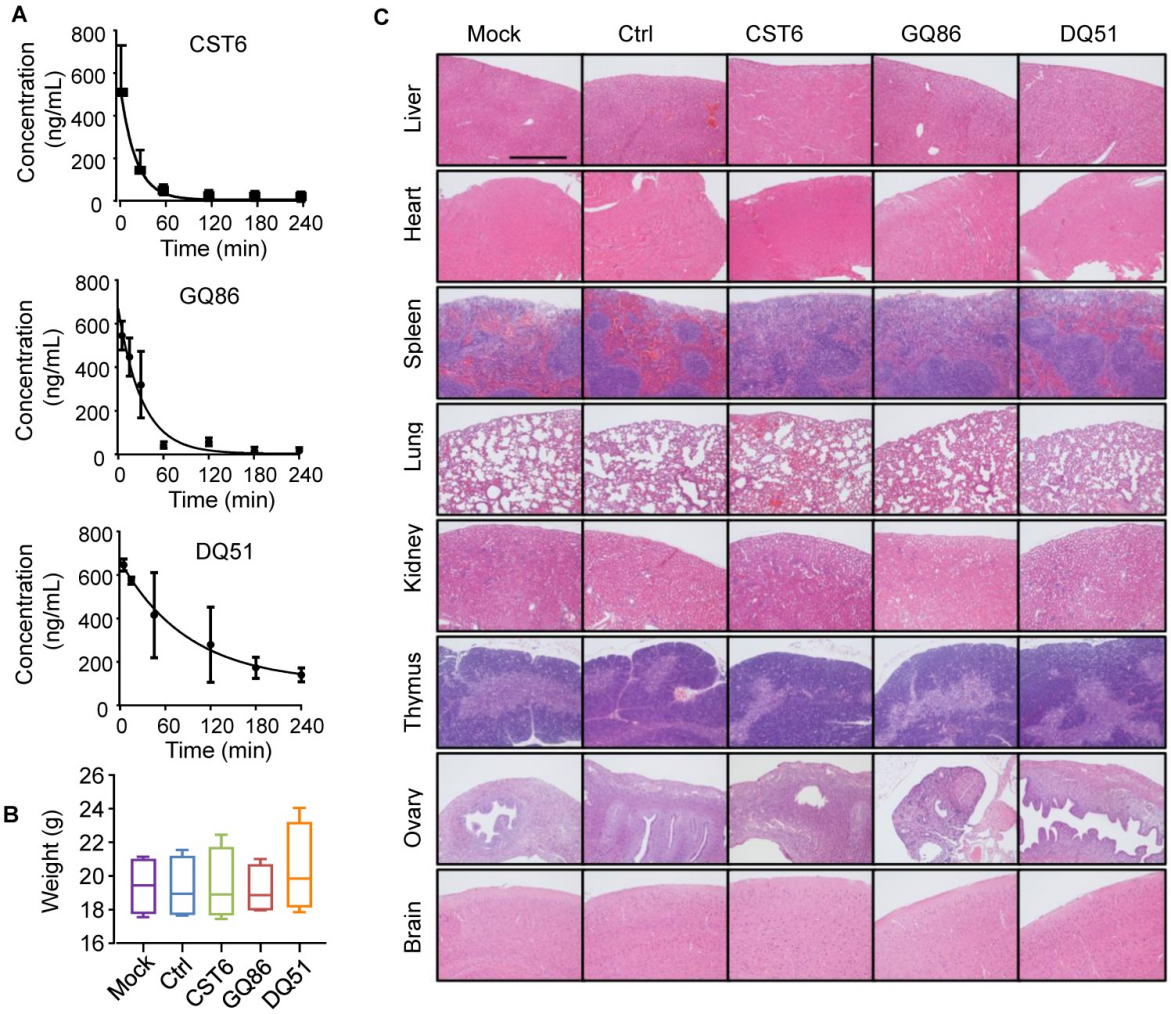
** Half-life and toxicity analysis of CST6 protein and peptides in mice.** (**A**) Half-life analysis of CST6 protein and peptides in the blood of mice. (**B**) Body weights of mice 4 weeks after daily treatment of 1 mg/kg/day CST6 protein and peptides (n = 4 mice per group). (**C**) H&E staining of various organs in mice 4 weeks after daily treatment of 1 mg/kg/day CST6 protein and peptides. Scale bar, 200 μm.

**Figure 7:**
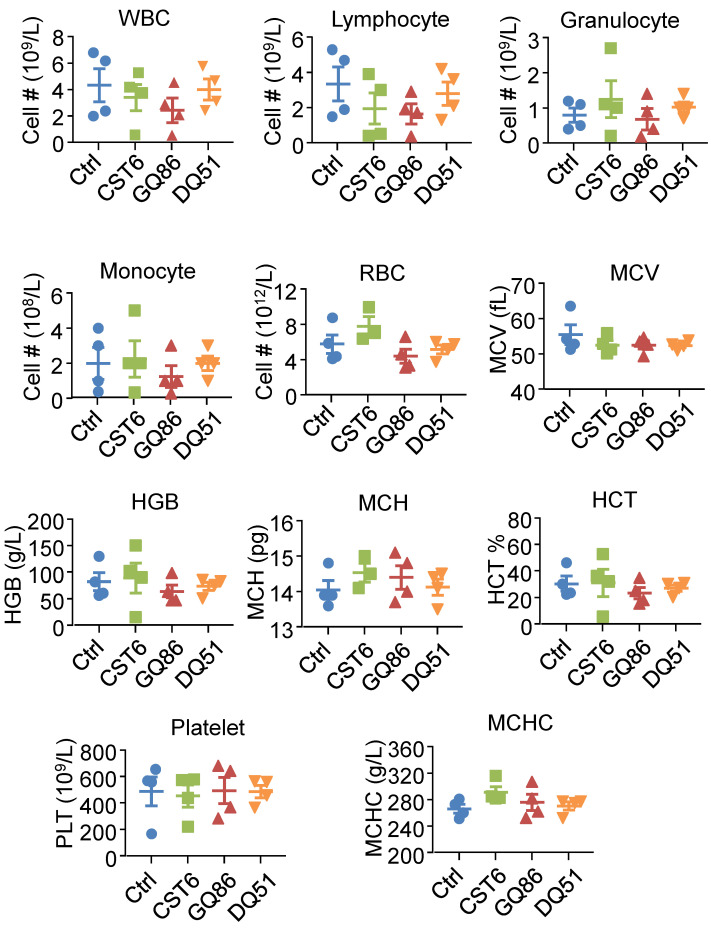
** Hematological analysis of mice after treatment of CST6 protein or peptides.** Shown are the hematological results after intravenous treatment of 1 mg/kg/day CST6 protein and peptides for 4 weeks. WBC, white blood cell; RBC, red blood cell; MCV, erythrocyte mean corpuscular volume; HGB, hemoglobin; MCH, mean corpuscular hemoglobin; HCT, red blood cell specific volume; MCHC, mean corpuscular hemoglobin concentration.

We then performed pharmacological evaluation of CST6 protein and peptides. Immunohistochemistry analyses demonstrated that the administrated protein mainly distributed in liver, kidney, intestine, spleen and bone metastasis foci of the mice (Fig. S6A). DQ51 displayed a longer plasma half-life than GQ86 and CST6 in the mice (Fig. 6A and Table S2). Acute toxicity analysis also showed that the maximum tolerance dose (MTD) of DQ51 (250 mg/kg) was slightly higher than that of GQ86 and CST6 (200 mg/kg). Importantly, MTD and median lethal dose (LD50, 322.00-360.10 mg/kg) of these candidate drugs (Table S3) were all significantly higher than the dose (1 mg/kg) that could effectively inhibit bone metastasis. Chronic toxicity assessment by daily intravenous administration of these peptides at the dose of 1 mg/kg revealed no apparent abnormality in the animals after 4 weeks. No differences were observed in the weights of whole body (Fig. 6B) or various organs including heart, liver, spleen, lung, kidney, adrenal gland, thymus, ovary and brain (Table S4) after the treatment. Histological examination of these organs revealed no abnormality either (Fig. 6C). Hematological analysis also showed that the treated mice appeared largely normal (Fig. 7). These data indicated the drug safety of CST6 and the related peptides, especially DQ51, for treating osteolytic bone metastasis.

## Discussion

Epigenetic silencing of CST6 has been widely observed in cancers 15, 28, 29 and it has long been implicated as a tumor suppressor. Indeed, previous studies have solidly demonstrated that CST6 suppresses the proliferation, survival 12, 30, 31 and metastasis of cancer cells 15. The functional role of CST6, as well as its relatively small size and the secretory nature, argues for the clinical potential of this protein in cancer treatment. However, the molecular mechanism of CST6 remained elusive, hindering the rational designing of CST6-based therapeutics. Here, our study elucidated a CST6-CTSB-SPHK1-p38 signal axis to regulate osteoclastogenesis and bone metastasis. We showed that CST6 targets CTSB to inhibit its enzymatic activity and stabilizes SPHK1 in osteoclasts. SPHK1 suppresses p38 activation and osteoclastogenesis, leading to disruption of the osteolytic vicious cycle of bone metastasis. Interestingly, our data showed that although the W135A mutation substituting the Tryptophan within the C-terminal hairpin loop of CST6 23-26 diminished its inhibitory effect on CTSB, several CST6 peptides without the loop fragment remained as CTSB inhibitors. These results suggest that the C-terminal loop may not be required for CST6 binding to CTSB. Instead, the W135A mutation might disrupt the protein structure and interfere CST6-CTSB binding. The exact role of this C-terminal loop, as well as that of the QLVAG domain, for CTSB inhibition is worthy of further investigation. In addition, it is not known yet how CST6-stabilized SPHK1 regulates p38 phosphorylation. Nevertheless, our study revealed a new role of CST6 to regulate tumor stroma instead of cancer cells. Importantly, extracellular CST6 protein can be internalized by osteoclasts through endocytosis to inhibit the CTSB activity, and the QLVAG fragment in the protein appears crucial for this inhibitory effect. These findings validate the potential of CST6-based approaches to treat bone metastasis and provide the rationale for screening of CST6 peptides as drug candidates.

Peptide drugs have demonstrated great potential in clinical application due to the advantages in potency, selectivity and low toxicity. Numerous peptide drugs, such as insulin, oxytocin, gonadotropin-releasing hormone, vasopressin and PTHrP, have been successful in pharmaceutical market 32. Our study also demonstrated the potential of several QLVAG-containing peptides to inhibit osteoclastogenesis. More importantly, the CST6 recombinant protein and peptides showed promising effects to treat *in vivo* bone metastasis of breast cancer. Although we could not directly compare the peptides to the clinical drug Denosumab in the animal model because the humanized antibody does not target bone resorption in mice 33, 34, the efficacy of the peptides is at least comparable to two other clinical drugs Zoledronic Acid and Bortezomib. In addition, our preliminary data of pharmacological analysis demonstrate that the MTD of these peptides in mice are significantly higher than the effective dose to inhibit bone metastasis and improve overall survival, indicating a broad therapeutic window of these candidate drugs. Long-term treatment with the peptides caused no apparent abnormality of the mice. In contrast, the chemical inhibitor CA-074, although also targeting CTSB and demonstrating a metastasis-inhibiting efficacy 35, caused apparent body weight loss of the mice (Fig. S6B), indicating a superior potential of peptide inhibitors of CTSB for metastasis treatment. However, plasma stability and oral bioavailability are still the main obstacles for development of peptide drugs. Our data indicated that shorter peptides appeared more stable without compromise of efficacy, thus providing the possibility of further optimization of the peptides with sequence screening and peptide modification. In addition, drug delivery vehicles could be also considered for controlled release of the peptides. Although bisphosphonates and Denosumab show positive impact on delay of metastasis progression, the effects of these osteoclast-targeting drugs to improve long-time patient survival have been minimal 36. In addition, resistance to these drugs also occurs. As the CST6 protein and peptides regulate osteoclastogenesis through a mechanism different to these clinical drugs, they represent promising candidates to supplement the current therapeutics of bone diseases.

## Materials and Methods

### Plasmids and reagents

The full length human *CST6* was cloned into the pLVX-puro vector (Clontech) for overexpression. Murine *Sphk1* was amplified from RAW264.7 cDNA and cloned into pMSCV (Clontech) vector for overexpression. For recombinant protein or peptide production in *E. coli*, full length or truncated CST6 sequences were subcloned into pET-28a (Novagen) with a C-terminal 6× His tag. The short hairpin RNAs (shRNAs) were cloned into the pLKO.1-puro vector (Addgene) for *CST6* or the pSuper-puro vector (Oligoengine) for *Sphk1*. The sequences of shRNAs for *CST6*, *Sphk1* and siRNAs for *Ctsb* and *Sphk1*, and qPCR primer sequences of *Caclr*, *Nfatc1*, *Acp5*, *Ctsk* and *Fos* were provided in Table S5. The CST6 mutants N64A (CST6^△N^) and W135A (CST6^△W^) were constructed as previously described 17. All constructs were confirmed by sequencing. The rabbit anti-CST6 polyclonal antibody (17076-1-AP, Proteintech), mouse anti-SPHK1 monoclonal antibody (365401, Santa Cruz), rabbit anti-phos-p38 (Thr180/Tyr182) polyclonal antibody (9211, Cell Signaling Technology) and rabbit anti-p38 polyclonal antibody (9212, Cell Signaling Technology) were used for Western blotting. Other reagents used in this study included CST6 neutralizing antibody (MAB1286, R&D), CA-074Me (HY-100350, Medchem Express), Z-FY(t-Bu)-DMK (219427, Merck), SB203580 (1076, Selleck) and recombinant mouse CST6 protein (1284, R&D).

### Cell culture

SCP2, SCP4 and RAW264.7 were maintained in Dulbecco's Modified Eagle's Medium (DMEM, HyClone) supplemented with 10% fetal bovine serum (FBS, Gibco), 100 units/ml penicillin and 100 μg/ml streptomycin (Gibco) in 5% CO_2_ at 37 °C.

### Fluorometric enzymatic assays

Enzymatic activities of cathepsins were analyzed with the fluorometric kits of CTSB (K147, Biovision) and CTSL (K161, Biovision) according to the manufacturer's protocol. The LGMN activity was analyzed as previously described 37 with minor modifications of the protocol. Briefly, the cells were harvested and kept in lysis buffer (100 mM sodium citrate, 1 mM disodium EDTA, 1% noctyl-beta-D-glucopyranoside, pH 5.8). After three freeze-thaw cycles at -80 °C, the cell lysate was centrifuged at 10000 g for 15 min and the supernatant was transferred into a new tube followed by protein quantification. 10 μg cell lysate in the assay buffer (39.5 mM citric acid, 121 mM Na_2_HPO_4_, 1 mM DTT, 1 mM EDTA, and 0.1% CHAPS, pH 5.8) in a total volume of 100 μL was added to the wells of a flat-bottom 96-well microplate (3631, Corning). After adding 1 μL substrate Z-Ala-Ala-Asn-AMC (I1865, Bachem) into each well with a final concentration of 10 μM, fluorescence measurements were performed every 5 min at 37 °C using a BioTek's Synergy™ Mx Microplate Reader with a 360 nm excitation filter and a 460 nm emission filter. All measurements were performed in triplicates.

### Production of recombinant proteins and peptides

CST6 wild type and mutant proteins, as well as the peptides longer than 30 amino acids, were expressed in BL21 (DE3) competent *E. coli* cells. The cells were transformed with the pET-28a plasmids expressing the protein or peptides, and then a single colony was transferred into 1 L of LB liquid medium with 100 μg/ml kanamycin. The cells were shaken at 37 ^o^C and 200 rpm until the absorbance reached 0.6 at 600 nm, followed by addition of 1 mM IPTG and incubation at 16 ^o^C for another 12 hours. The cells were harvested by centrifugation at 5000 rpm for 15 min and the pellet was resuspended in lysis buffer (50 mM NaH_2_PO_4_, 0.3 M NaCl, pH 8.0). After treatment with 1 mg/mL Lysozyme, the cells were sonicated for 60 s and the lysate was centrifuged at 12,000 g for 30 min. The clear supernatant was collected and filtered with a 0.45-μm syringe filter. The purification was performed with the HisSep Ni-NTA 6FF Chromatography Column (20504, Yeasen Biotech) according to manufacturer's instruction. The target proteins were visualized by Western blot analysis and Coomassie staining.

Peptides of 30 amino acids or shorter were chemically synthesized and purchased from Ontores Biotech (GM30) and GL Biochem (AY11 and DR9). All peptides were obtained with a purity > 95%.

### Osteoclastogenesis assays

Primary bone marrow cells were harvested by flushing the cavity of tibia and femur from 5-6 week old Balb/c mice. Erythrocytes were removed by treating cell suspension with the Red Blood Cell Lysing Buffer (R7757, Sigma) for 5 min. The remaining cells were cultured in α-MEM (A1049001, Gibco) supplemented with 10% FBS and 5 ng/ml M-CSF for 12 h. Non-adherent cells were transferred into a 24-well plate and cultured in α-MEM supplemented with 10% FBS, 25 ng/ml M-CSF and 25 ng/ml RANKL for another 5 to 6 days. Cancer cell CM was mixed with α-MEM at a ratio of 1:3. CST6 protein, peptides or other drugs at indicated concentrations were administrated into the culture medium. For assessment of osteoclast differentiation, cells were stained using the Acid Phosphatase TRAP staining kit (387A, Sigma) as previously described 38. For RAW264.7 osteoclastogenesis assay, 700 cells per well were added into a 96-well plate and cultured in the same medium as shown above except that M-CSF was not essential. 7 ug/ml CST6 antibody (MAB1286, R&D) was used to neutralize recombinant human CST6 in RAW264.7 osteoclastogenesis assay.

### Mouse experiments of bone metastasis

For bone metastasis study was performed as previously described 38. Briefly, athymic Balb/c mice were anesthetized and 10^5^ cells in 100 μL phosphate-buffered saline (PBS) were injected into the left cardiac ventricle. Peptides were administrated intravenously into the mice (n = 10 per group) at a dose of 1 mg/kg/day. The control mouse group were administered with same volume of peptide solvents. The metastatic burden was measured by bioluminescent imaging (BLI) with a NightOWL II LB983 Imaging System (Berthold). The osteolytic area was analyzed by the SkyScan 1076 *In vivo* X-ray Microtomograph (Bruker). The sections of hind legs of the mice was used for H&E, TRAP and immunohistochemistry staining.

### Pharmacokinetic and toxicity assays

For pharmacokinetic assays, CST6 protein or His-tagged peptides were administrated intravenously into Balb/c mice (n = 4 per group) at a dose of 1 mg/kg. Blood samples (100 μL) were collected using capillary at 0.25 h, 0.5 h, 1 h, 2 h, 3 h and 4 h, respectively. After 1 h at room temperature, the serum samples were obtained from the supernatant by centrifugation at 3000 rpm for 15 min. The CST6 content in serum was analyzed with CST6 ELISA kit (Sino Biological, 10438). The peptide content was analyzed with the His-tag ELISA kit (GenScript, L00436) according to the manufacturer's instruction. Blank serum samples from mice without protein/peptide administration were used as control. Standard curves for each protein or peptide were generated with standard samples of known concentrations to calculate the protein/peptide concentration in blood samples. The data for all peptides were fitted with the one-phase decay model using Prizm software (GraphPad Software) to calculate the half-lives.

For acute toxicity assays, Balb/c mice (n = 5 per group) were intravenously injected with the indicated protein or peptides at doses of 150, 200, 250, 300, 350 mg/kg. The death of mice was counted after 24 h. The median lethal dose (LD50) was calculated using the Bliss method. Acute toxicity assays were repeated three times, and all analyses showed similar results.

For chronic toxicity assays, Balb/c mice (n = 4 per group) were treated with daily intravenous injection of the indicated protein or peptides at a dose of 1 mg/kg/day for 4 weeks, or 10 mg/kg/day CA-074 for 8 days. The body weights of the mice were measured. After 4 weeks of CST6 peptide treatment, blood samples (50 μL) were collected using tubes and immediately diluted by PBS containing 5 mM EDTA into 100 μL, and analyzed by an Auto Hematology Analyzer (Mindray, BC-2800 Vet). Mice were sacrificed and organs were harvested for weight measurements and H&E staining to observe the pathological changes.

### Statistical analysis

BLI curves of *in vivo* bone metastasis were compared by two-tailed nonparametric Mann-Whitney test without assumption of Gaussian distribution. Log-rank test was performed for survival analyses of the mice. Two-tailed independent Student's t-test without assumption of equal variance was performed to analyze the *in vitro* and *in vivo* osteoclastogenesis, as well as other assays. *P* values less than 0.05 were considered as significant.

## Supplementary Material

Supplementary figures and tables.
