# Supplementary material for: CST6 protein and peptides inhibit breast cancer bone metastasis by suppressing CTSB activity and osteoclastogenesis
Source: Theranostics. 2021 Oct 11;11(20):9821–32. doi: 10.7150/thno.62187 (PMC8581426; doi:10.7150/thno.62187)
Supplement: Supplementary file 1 — Supplementary figures and tables. [file thnov11p9821s1.pdf]

Supplementary Information

Supplementary Information contains 6 Supplementary Figures and 5 Supplementary Tables. In addition, uncropped images of all Western blots in the figures were attached as Supplementary Fig. 7.

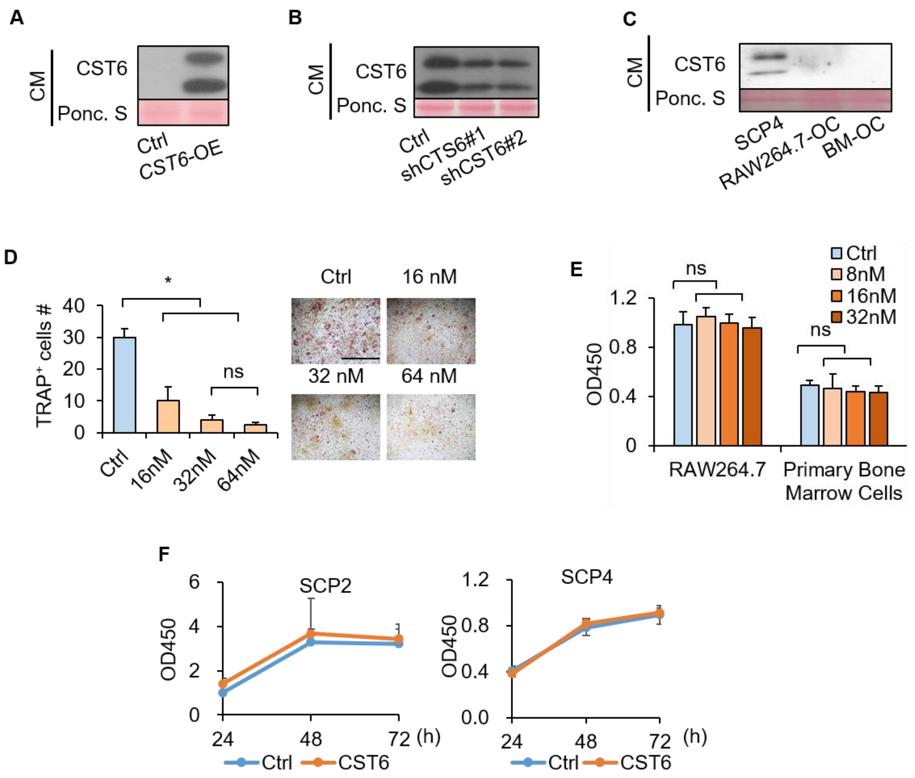

Supplementary Figure 1. The effects of CST6 on osteoclasts and tumor cells.

(**A**) CST6 secretion level in SCP2 cells after overexpression. Note that the two bands of CST6 represent the glycosylated (17 kDa) and unglycosylated (14.4 kDa) forms. Ponc. S, Ponceau S. (**B**) CST6 secretion level in SCP4 cells after knockdown. (**C**) Western blot analysis of secreted CST6 in SCP4 and RAW264.7 or primary bone marrow (BM)-derived osteoclasts (OC). (**D**) Osteoclastogenesis of murine primary bone marrow cells with different concentrations of recombinant CST6 protein (n = 3 independent experiments). Scale bar, 200 μm. (**E**) Viability of RAW264.7 or primary bone marrow-derived osteoclasts after CST6 recombinant protein treatment (n = 4 independent experiments). (**F**) SCP2/SCP4 tumor cell growth after CST6 recombinant protein treatment (32 nM); n = 4 biological repeats.

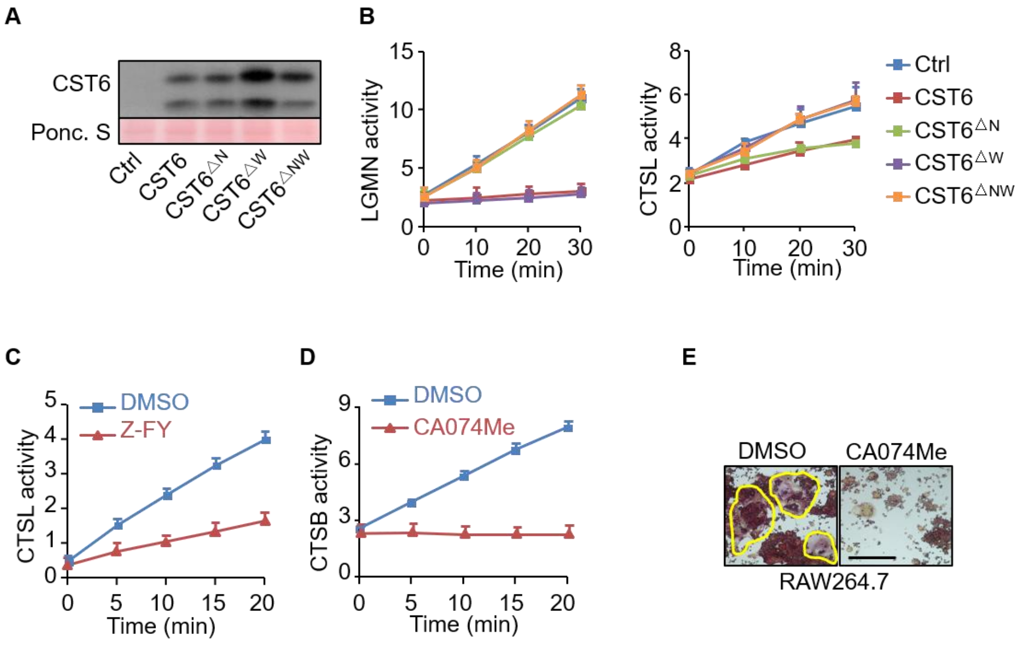

Supplementary Figure 2. The effects of CST6 mutants and cathepsin inhibitors on enzymatic activities of LGMN, CTSL and CTSB.

(**A**) Western blot analysis of secreted CST6 mutants in SCP2 after overexpression. (**B**) LGMN and CTSL enzymatic activity assay in SCP2 after CST6 mutant overexpression. (**C**) CTSL enzymatic activity assay of RAW264.7 after treatment with 10 μM Z-FY(t-Bu)-DMK (Z-FY). (**D**) CTSB enzymatic activity assay in RAW264.7 after treatment with 10 μM CA-074Me. (**E**) Representative images of RAW264.7 osteoclastogenesis after treatment with CA074Me. Yellow circles denote mature osteoclasts.

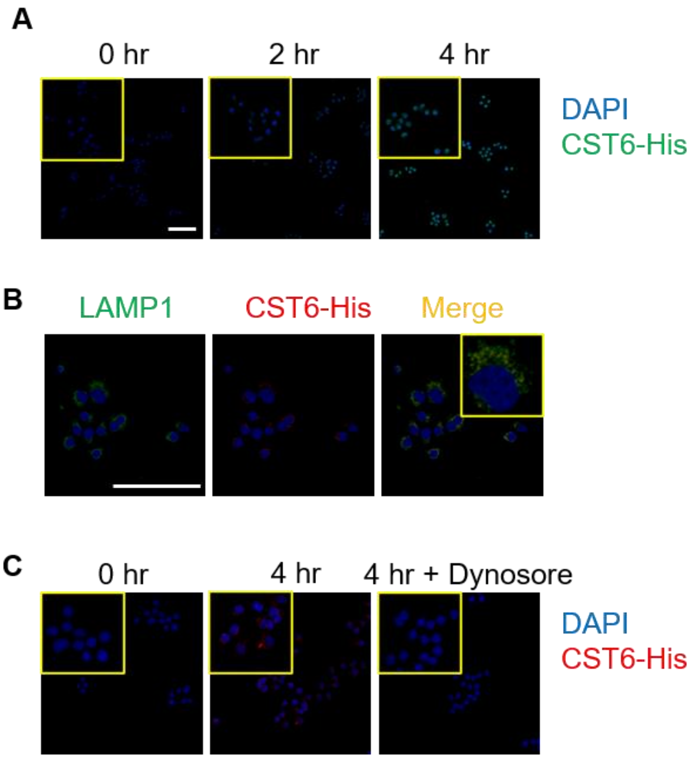

Supplementary Figure 3. Immunofluorescent (IF) analyses of internalized CST6 protein in primary bone marrow-derived osteoclasts.

(**A**) IF of CST6 in osteoclasts after culturing osteoclasts with 32 nM His-tagged recombinant CST6 protein for various time. (**B**) IF of intracellular CST6-His and the lysosome marker LAMP1 after culturing osteoclasts with 32 nM CST6-His protein. (**C**) IF of recombinant CST6-His in osteoclasts after culturing osteoclasts with CST6-His and Dynosore (30 μM). Scale bar, 150 μm.

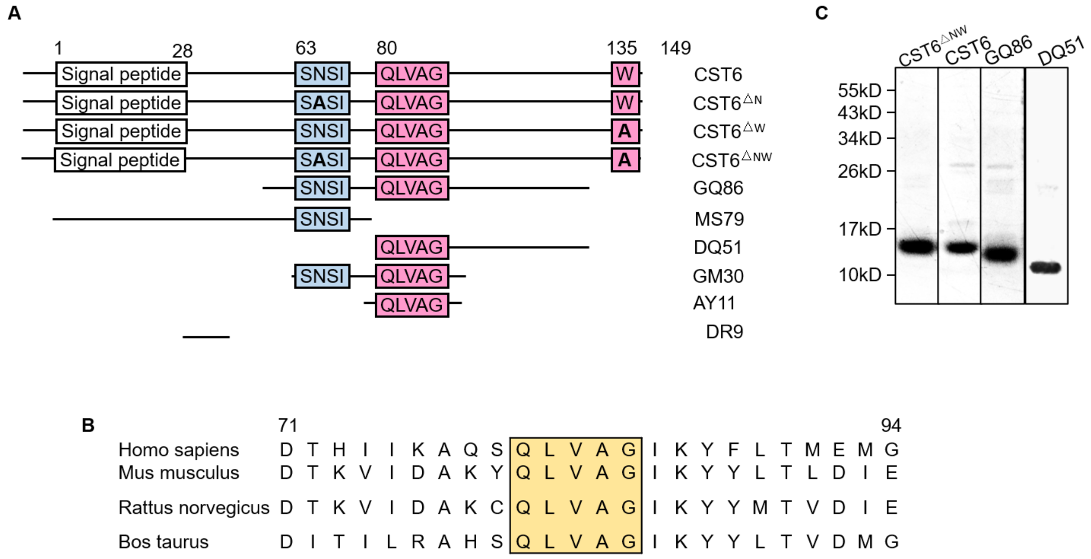

Supplementary Figure 4. The designing and production of CST6 peptides. (**A**) Schematic diagram of the structures of CST6 protein and peptides. The QLVAG fragment and the C-terminal W^{135} that are critical for CST6 binding to CTSB[1-5] are highlighted in red, while the SNSI fragment critical for binding to LGMN[1, 6] is highlighted in blue. (**B**) Conservation of the QLVAG fragment of CST6 in different species. (**C**) Coomassie blue staining of CST6 proteins and peptides after recombinant expression and purification.

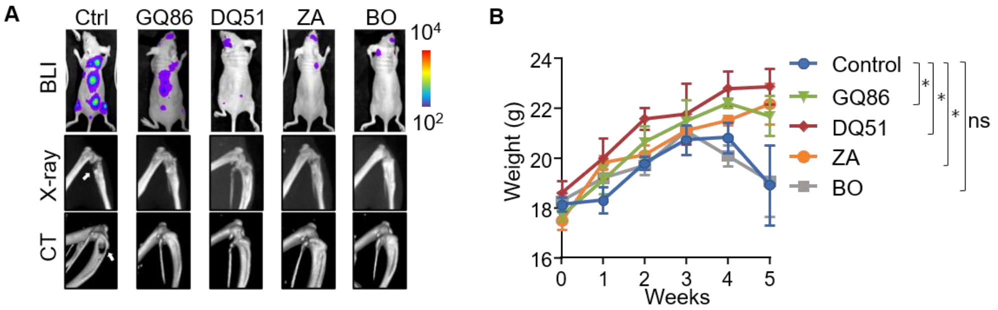

Supplementary Figure 5. The effects of CST6 peptides and clinical drugs to inhibit *in vivo* bone metastasis.

(**A**) SCP2 bone metastasis in nude mice with treatment of 1 mg/kg/day CST6 peptides, 1 mg/kg/day Zoledronic Acid (ZA) or 1 mg/kg/day Bortezomib (BO). Shown are representative BLI, X-ray and micro-CT images at week 4 of treatment. (**B**) Body weights of the mice at week 5.

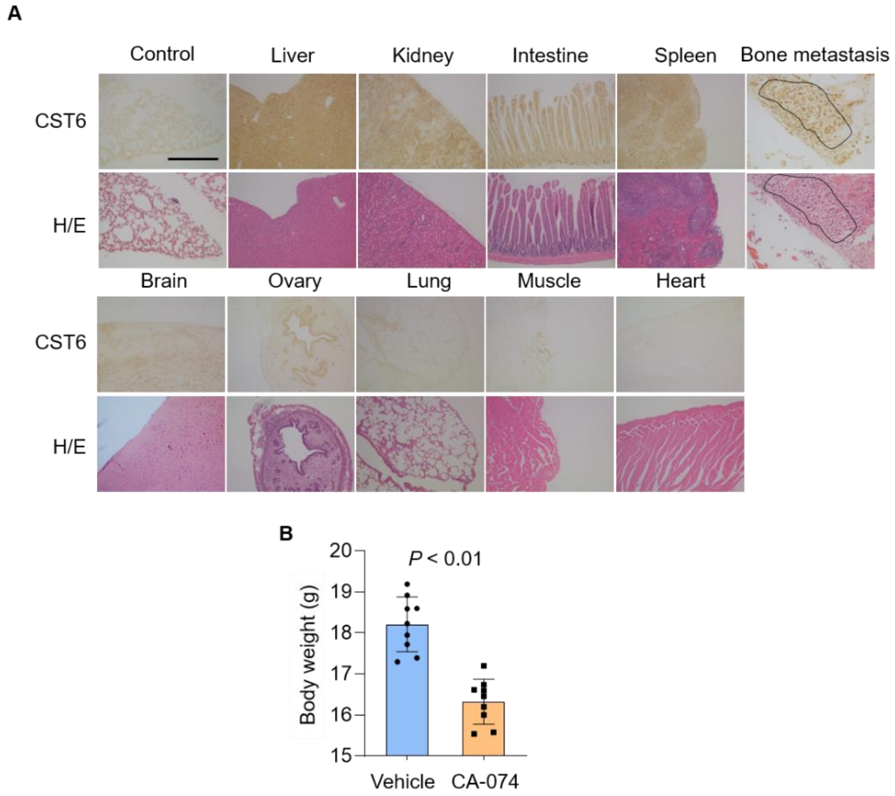

Supplementary Figure 6. Pharmacological evaluation of CST6 recombinant protein and CA-074.

(**A**) CST6 immunohistochemistry in various organs at week 5 after administration of 1 mg/kg/day CST6 peptides. The lung section from un-treated mice was used as negative control of the assay. Dotted lines denotes areas of bone metastasis. Scale bar, 150 μm. (**B**) The body weights of nude mice after treatment of 10 mg/kg CA-074 for 8 consecutive days (n = 9 mice per groups).

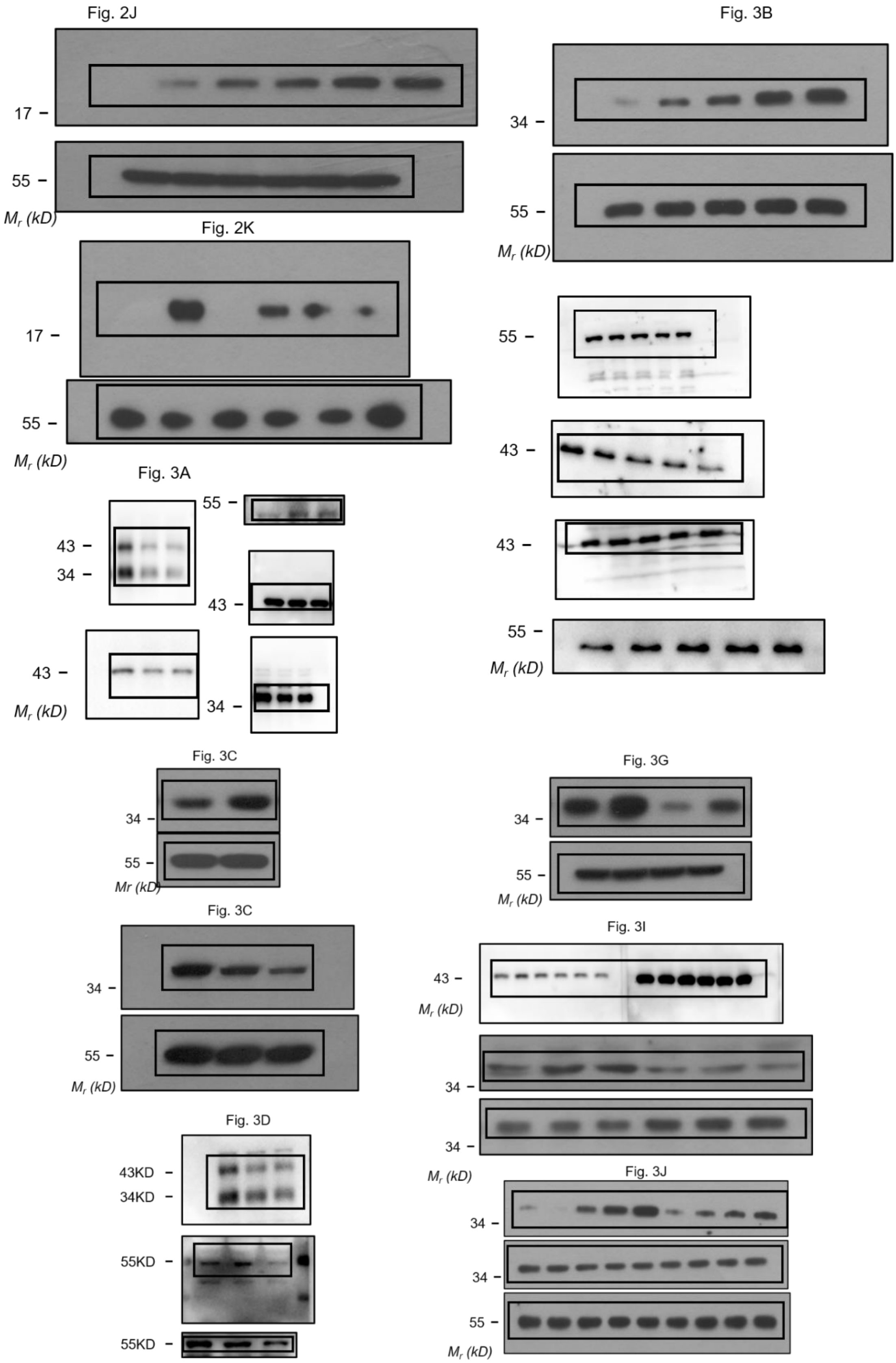
Fig. 2J
17
55
Mr (kD)
Fig. 2K
17
55
Mr (kD)
Fig. 3A
43
34
55
43
43
Mr (kD)
34
Fig. 3B
34
55
Mr (kD)
55
43
43
55
Mr (kD)
Fig. 3C
34
55
Mr (kD)
Fig. 3C
34
55
Mr (kD)
Fig. 3D
43KD
34KD
55KD
55KD
Fig. 3G
34
55
Mr (kD)
Fig. 3I
43
Mr (kD)
34
34
Mr (kD)
Fig. 3J
34
34
55
Mr (kD)

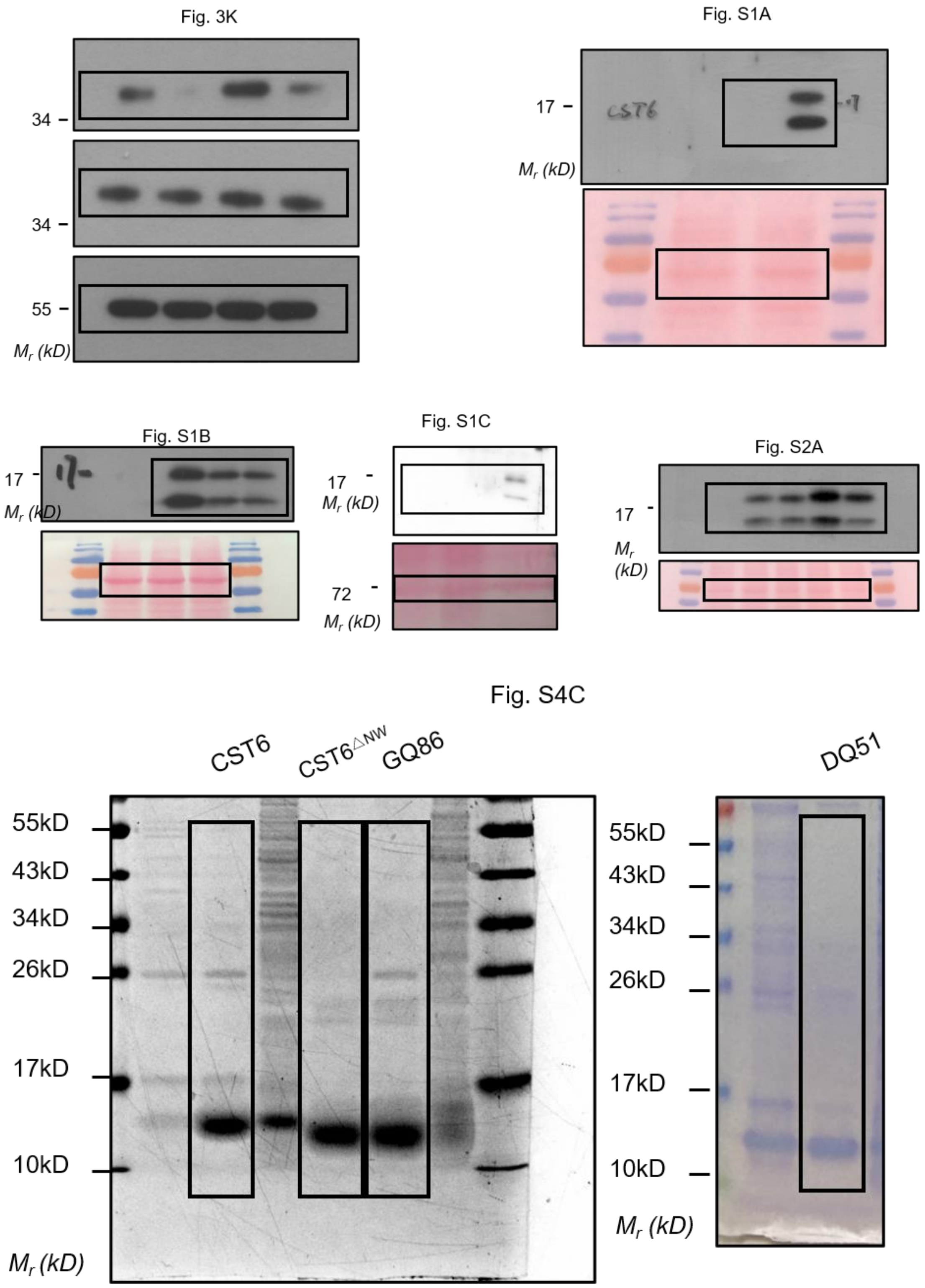

Supplementary Figure 7. Uncropped images of Western blots.

Supplementary Tables:

Table S1. Amino acid sequences of CST6-related peptides.

Name	Length	Sequence
CST6	149	MARSNLPLALGLALVAFCLLALPRDARARPQERMVGELRDLSPDDPQVQKAAQAAVASYNM<br>GSNSIYYFRDTHIIKAQSQLVAGIKYFLTMEMGSTDCRKTRVTGDHVDLTTCPLAAGAQQEKL<br>RCDFEVLVVPWQNSSQLLKHNCVQM
CST6△N	149	MARSNLPLALGLALVAFCLLALPRDARARPQERMVGELRDLSPDDPQVQKAAQAAVASYNM<br>GSASIYYFRDTHIIKAQSQLVAGIKYFLTMEMGSTDCRKTRVTGDHVDLTTCPLAAGAQQEKL<br>RCDFEVLVVPWQNSSQLLKHNCVQM
CST6△W	149	MARSNLPLALGLALVAFCLLALPRDARARPQERMVGELRDLSPDDPQVQKAAQAAVASYNM<br>GSNSIYYFRDTHIIKAQSQLVAGIKYFLTMEMGSTDCRKTRVTGDHVDLTTCPLAAGAQQEKL<br>RCDFEVLVVPAQNSSQLLKHNCVQM
CST6△NW	149	MARSNLPLALGLALVAFCLLALPRDARARPQERMVGELRDLSPDDPQVQKAAQAAVASYNM<br>GSASIYYFRDTHIIKAQSQLVAGIKYFLTMEMGSTDCRKTRVTGDHVDLTTCPLAAGAQQEKL<br>RCDFEVLVVPAQNSSQLLKHNCVQM
GQ86	86	GELRDLSPDDPQVQKAAQAAVASYNMGSNSIYYFRDTHIIKAQSQLVAGIKYFLTMEMGSTD<br>CRKTRVTGDHVDLTTCPLAAGAQQ
MS79	79	MARSNLPLALGLALVAFCLLALPRDARARPQERMVGELRDLSPDDPQVQKAAQAAVASYNM<br>GSNSIYYFRDTHIIKAQS
DQ51	51	DTHIIKAQSQLVAGIKYFLTMEMGSTDCRKTRVTGDHVDLTTCPLAAGAQQ
GM30	30	GSNSIYYFRDTHIIKAQSQLVAGIKYFLTM
AY11	11	AQSQLVAGIKY
DR9	9	DARARPQER

Table S2. Circulation half-lives of CST6 peptides in mice.

Group (n=4)	Model	Half-life (min)	95% confidence intervals (min)
CST6	One-phase decay	10.48	8.23-14.38
GQ86	One-phase decay	20.58	13.14-33.91
DQ51	One-phase decay	62.75	47.72-168.20

Table S3. LD50 and MTD of CST6 peptides in mice.

Protein/Peptide	LD50 (mg/kg)	MTD (mg/kg)
CST6	322.00±117.10	200
GQ86	377.70±146.05	200
DQ51	360.10±74.25	250

Table S4. Organ weight measurements for chronic toxicity analysis.

Group (n=4)		Mock	Ctrl	CST6	GQ86	DQ51
Heart	Weight (g)	0.125±0.025	0.128±0.013	0.123±0.010	0.125±0.006	0.130±0.014
	p- value compared to Ctrl	0.865	-	0.550	0.730	0.800
Liver	Weight (g)	1.028±0.093	1.155±0.237	1.065±0.298	1.098±0.114	1.230±0.057
	p- value compared to Ctrl	0.355	-	0.653	0.677	0.561
Spleen	Weight (g)	0.108±0.01	0.105±0.026	0.110±0.024	0.123±0.015	0.135±0.019
	p- value compared to Ctrl	0.865	-	0.791	0.294	0.116
Lung	Weight (g)	0.195±0.076	0.140±0.014	0.145±0.021	0.140±0.017	0.155±0.035
	p- value compared to Ctrl	0.204	-	0.705	0.999	0.458
Kidney	Weight (g)	0.285±0.041	0.248±0.028	0.288±0.052	0.287±0.023	0.288±0.026
	p- value compared to Ctrl	0.181	-	0.222	0.104	0.080
Adrenal Gland	Weight (g)	0.011±0.001	0.028±0.017	0.055±0.070	0.011±0.001	0.018±0.010
	p- value compared to Ctrl	0.098	-	0.477	0.098	0.346
Thymus	Weight (g)	0.053±0.031	0.053±0.029	0.065±0.010	0.038±0.028	0.028±0.029
	p- value compared to Ctrl	0.999	-	0.443	0.479	0.264
Ovary	Weight (g)	0.163±0.097	0.155±0.137	0.238±0.151	0.190±0.063	0.205±0.151
	p- value compared to Ctrl	0.932	-	0.448	0.658	0.641
Brain	Weight (g)	0.385±0.054	0.398±0.046	0.438±0.026	0.435±0.007	0.403±0.066
	p- value compared to Ctrl	0.721	-	0.138	0.267	0.899

Table S5. Sequences of qPCR primers and shRNA/siRNA constructs.

	Gene	Sequence
qPCR primers	Gapdh-F	TGTCCGTCGTGGATCTGAC
	Gapdh-R	CCTGCTTCACCACCTTCTTG
	Calcr-F	GGTTCCTTCTCGTGAACAGGT
	Calcr-R	GGTAGGAGCCTGAAGAACTGG
	Nfatc1-F	TCCAAAGTCATTTTCGTGGA
	Nfatc1-R	CTTTGCTTCCATCTCCCAGA
	Acp5-F	CGTCTCTGCACAGATTGCAT
	Acp5-R	AAGCGCAAACGGTAGTAAGG
	Ctsk-F	CTCCATCGACTATCGAAAGAAAG
	Ctsk-R	AAAGCCCAACAGGAACCAC
	Fos-F	GGGACAGCCTTTCCTACTACC
	Fos-R	AGATCTGCGCAAAAGTCCTG
shRNAs /siRNAs	shCST6#1	GCTGCGCTGTGACTTTGAGGT
	shCST6#2	CTTCCGAGACACGCACATCAT
	shSphk1#1	CCTTCCAGTTAGAGTAACA
	shSphk1#2	TATGGAACTTGACTGTCCA
	siControl	UUCUCCGAACGUGUCACGU
	siCtsb#1	GCUGUCGGAUGACCUGAUU
	siCtsb#2	GGACAUAGAUCUACCUGAA
	siSphk1	GAGGCAGAGAUAACCUUUA

References of Supplementary Information:

1. Dall, E., J.C. Fegg, P. Briza, and H. Brandstetter, Structure and mechanism of an aspartimide-dependent peptide ligase in human legumain. Angew Chem Int Ed Engl, 2015. **54**(10): 2917-21.

2. Ni, J., M. Abrahamson, M. Zhang, M.A. Fernandez, A. Grubb, J. Su, et al., Cystatin E is a novel human cysteine proteinase inhibitor with structural resemblance to family 2 cystatins. J Biol Chem, 1997. **272**(16): 10853-8.

3. Schuttelkopf, A.W., G. Hamilton, C. Watts, and D.M. van Aalten, Structural basis of reduction-dependent activation of human cystatin F. J Biol Chem, 2006. **281**(24): 16570-5.

4. Matsumoto, K., M. Murata, S. Sumiya, K. Mizoue, K. Kitamura, and T. Ishida, X-ray crystal structure of papain complexed with cathepsin B-specific covalent-type inhibitor: substrate specificity and inhibitory activity. Biochim Biophys Acta, 1998. **1383**(1): 93-100.

5. Musil, D., D. Zucic, D. Turk, R.A. Engh, I. Mayr, R. Huber, et al., The refined 2.15 A X-ray crystal structure of human liver cathepsin B: the structural basis for its specificity. Embo J, 1991. **10**(9): 2321-30.

6. Martinez, M., M. Diaz-Mendoza, L. Carrillo, and I. Diaz, Carboxy terminal extended phytocystatins are bifunctional inhibitors of papain and legumain cysteine proteinases. FEBS Lett, 2007. **581**(16): 2914-8.
